# The impact of environmental factors on phenotypic diversity of natural populations of *Polyspora* in China

**DOI:** 10.3389/fpls.2025.1553671

**Published:** 2025-10-10

**Authors:** Changle Ma, Maiyu Gong, Qing Gui, Zhifeng Fan, Jianxin Yang, Lijuan Wang, Lilan Deng

**Affiliations:** ^1^ College of Landscape Architecture and Horticulture Sciences, Southwest Forestry University, Kunming, China; ^2^ Southwest Research Center for Engineering Technology of Landscape Architecture (State Forestry and Grassland Administration), Kunming, Yunnan, China

**Keywords:** *Polyspora*, phenotypic traits, genetic factors, geoecological factors, natural populations

## Abstract

**Background:**

Plant phenotypic diversity is not solely determined by genetic variation but is also shaped by the combined effects of environmental factors. Polyspora, a genus within the Theaceae family, consists of evergreen trees or shrubs widely recognized for their horticultural value and suitability for afforestation in mountainous regions. Despite its ecological and economic significance, the genus Polyspora has received relatively limited attention from the plant taxonomy community, and no systematic studies on its phenotypic diversity have been conducted to date.

**Methods:**

Thus, we conducted a comprehensive investigation on the phenotypic traits of Polyspora (8 species, 32 populations) distributed across China. We employed nested variance analysis to characterize the variation patterns of phenotypic traits within and among populations. Furthermore, redundancy analysis and Pearson correlation analysis were conducted to explore the relationships between leaf morphological traits and geo-environmental variables. A phylogenetic tree was constructed based on 15 morphological traits, and the strength of the phylogenetic signal was quantified using Blomberg’s K statistic.

**Results:**

The results indicate that the phenotypic traits of Polyspora species exhibit significant interspecific and intraspecific differences, with abundant phenotypic variation. Specifically, the average coefficients of variation (CVs) were 24% for leaf traits, 11.98% for floral traits, and 17.49% for fruit and seed traits. In terms of variation degree, P. longicarpa exhibited the highest variation in leaf traits, P. chrysandra showed the greatest variation in floral traits, and P. axillaris displayed the maximum variation in fruit and seed traits. Among these species, P. speciosa had the largest leaves, whereas P. longicarpa possessed the largest flowers, fruits, and seeds. The 15 morphological traits examined, including style length, sepal length, capsule length, and seed wing length exhibited strong phylogenetic signals (K >>1, P< 0.05). Among the environmental factors analyzed, bioclimatic variables and ultraviolet radiation were found to exert a significant influence on leaf trait variation.

**Conclusions:**

These findings improve our understanding of the morphological characteristics of Polyspora leaves and the extent to which environmental factors drive phenotypic variation. Furthermore, this study provides a scientific basis for the conservation and sustainable utilization of Polyspora resources in future research and practical applications.

## 1 Introduction

Phenotypic analysis involves the examination of plant traits based on morphology and constitutes a crucial component in the study of plant genetic diversity (Khadivi et al., 2019). Through comprehensive exploration of phenotypic traits, it becomes possible to identify valuable plant resources and effectively utilize existing germplasm. Plant phenotypic traits can directly reflect the genetic characteristics of plants, thereby providing researchers with intuitive information for studies (Costa et al., 2017). In this process, leaves are important organs for plants to perform photosynthesis and transpiration (Chapin et al., 1993). Phenotypic changes in leaves can indicate the extent of environmental impact on plants. Given the significant morphological differences observed in plant leaves across various growth environments, these characteristics naturally serve as an important foundation for interspecific classification and analysis of phenotypic variation. Furthermore, floral phenotypic traits play a crucial role in evaluating plant germplasm resources and are considered traditional characteristics for the complex phenotypic identification of different taxonomic groups as well as for assessing the interactions between evolutionary and developmental variations (Wessinge and Hileman, 2016). The data derived from floral morphological characteristics encompass a substantial array of qualitative and quantitative trait information; thus, phenotypic diversity analysis is deemed the most appropriate tool for its evaluation (Khadivi-khuband and Anjam, 2014; Oliveira et al., 2012). Plant fruit and seed traits represent relatively stable genetic characteristics; however, there exhibit notable variation in phenotypes among seeds sourced from different provenances. These variations arise from long-term adaptations of plants to their natural environments along with their respective phenologies. Consequently, by analyzing the morphological features across various aspects such as leaves, flowers, fruits, and seeds, we can gain deeper insights into the genetic basis underlying plant phenotypic traits—providing a scientific foundation for advancements in plant breeding and genetic improvement.

The genus *Polyspora* belongs to the Theaceae family, which was established by Sweet in 1826. It consists of 47 species, which are evergreen shrubs or trees with a rich variety of flower colors. They are distributed in South and Southeast Asia, primarily found in countries such as Malaysia, Indonesia, China, and Vietnam. In the “Red List of Theaceae,” six species of *Polyspora* are listed as critically endangered, three species are listed as vulnerable, and four species are listed as near threatened (Beech et al., 2017). *Polyspora* plants are evergreen broad-leaved tree species, with domestic varieties being trees or shrubs.

The genus *Polyspora* is outstanding for its ornamental traits, it is a group of excellent garden landscape tree species that are known mainly for their winter flowering, while also offering attractive foliage, fruit, trunk, and tree shape. The genus *Polyspora*, as a widely distributed native tree species in southern China, is currently cultivated only in a few botanical gardens within the country and has sporadic applications in some cities. The plants of the genus *Polyspora* have characteristics such as rapid growth, strong adaptability, high water content in leaves, round and full tree crowns, straight trunks, and dense wood texture. Most species are able to adapt to barren mountainous areas and are excellent mountain afforested and timber tree species in tropical and subtropical regions. The fruits of *Polyspora* plants often contain natural antioxidants, and the extracts from the roots and stems have high cytotoxic activity (Li et al., 2019a; Fu et al., 2013; Tang, 2013). These extracts can be developed into functional foods or nutritional health products, which have the ability to prevent certain diseases caused by oxidative stress, such as cardiovascular diseases and cancer. However, due to the late start of basic research, insufficient nursery cultivation, and market development, only a few species of *Polyspora* plants in China have sporadic applications in limited areas. The vast majority of species remain in the wild and have not yet been rationally developed and utilized.

Therefore, based on a nationwide survey of *Polyspora* resources, this study investigates the phenotypic traits of 32 wild populations across 8 species. By studying the phenotypic diversity and the correlation between leaf phenotypes and geographical environmental factors. The objectives of this work are to elucidate how leaf phenotypes adapt to different environments, identify morphological features that carry phylogenetic signals, and thereby establish a foundation for subsequent analyses of molecular genetic diversity.

## 2 Materials and methods

### 2.1 Sample collection

**Figure 1:**
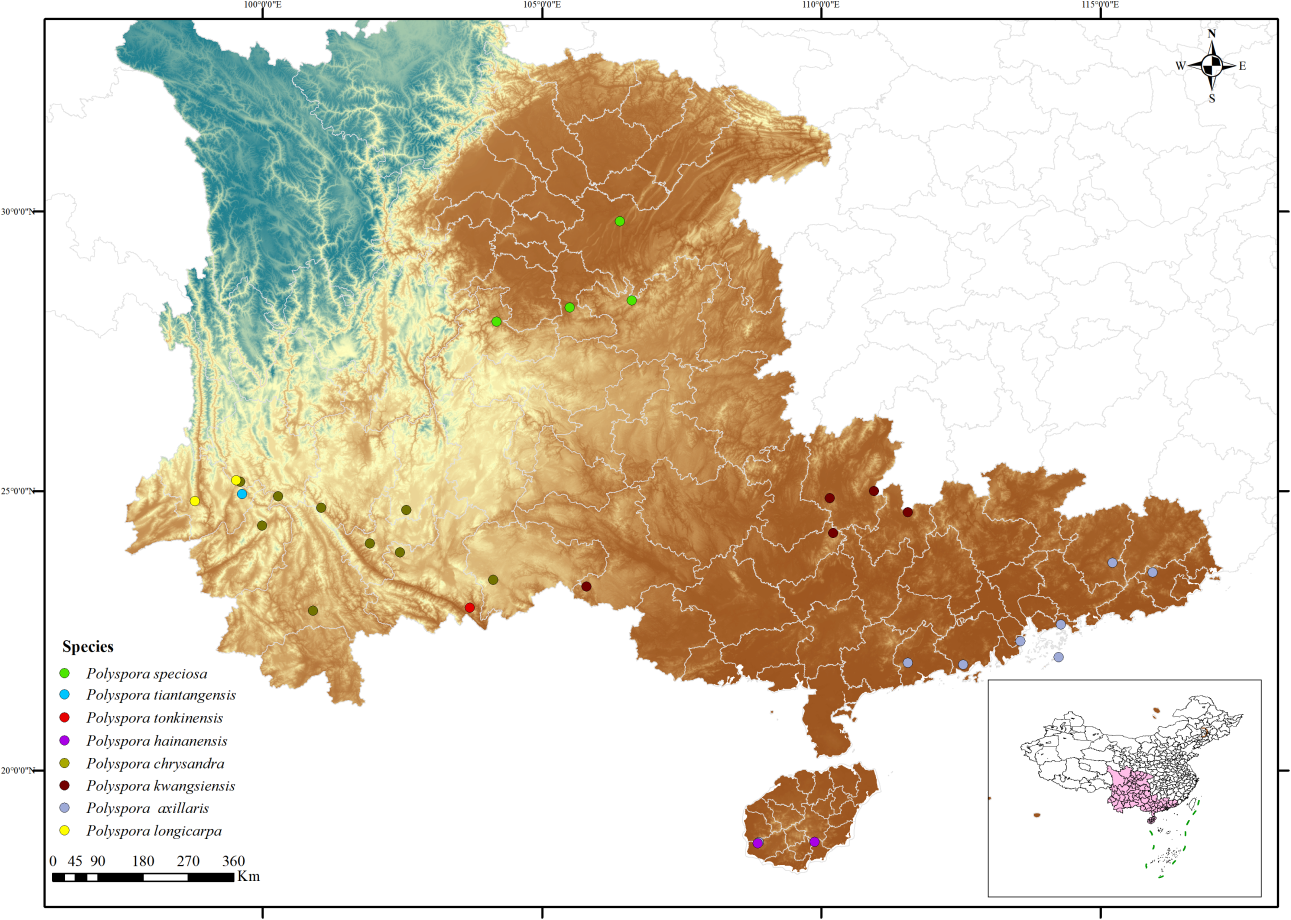
Distribution map of sampling sites for the natural population of *Polyspora.* Based on the standard map service website of the Ministry of Natural Resources with the approval number GS (2022) 1873, and the boundary of the base map has not been modified.

Based on the distribution of the *Polyspora* genus across the country, provinces with relatively high concentrations of *Polyspora* species and individuals, such as Yunnan, Guizhou, Sichuan, Chongqing, Guangxi, Guangdong, and Hainan, were selected to determine the scope and locations of the survey (**Figure 1**). According to factors such as different altitudes, habitats, and community structures, 1–2 representative populations in each survey area were identified. When there is no obvious geographical isolation such as rivers, valleys, or gullies, the minimum population distance for the same species should be restricted to more than 50 km; when there is obvious geographical isolation, the population distance is not limited.

Within each population, more than 20 samples of *Polyspora* plants were selected, with an individual spacing of more than 50 m. If the population sample size is fewer than 20 individuals or if there are obvious phenotypic differences within the population, the sampling is not limited by population size and sampling interval, and all individuals are collected. From each population, individuals with obvious phenotypic differences and stable traits were selected, some morphological data of leaves and flowers in the field were measured. The collected fruits were stored in sealed plastic bags with ice packs for preservation and sent back to the laboratory for actual measurement of their morphological data. After the fruits were dried and split open, the morphological data of the seeds were collected.

From 2020 to 2022, a total of 753 individual materials (**Supplementary Table S1**) from 32 populations of *Polyspora* in China were collected. The average distance between populations was 744 km, with more than 100 samples collected, as well as a small amount of pollen and fruit. This included 8 populations of *Polyspora axillaris*, 9 populations of *Polyspora chrysandra*, 5 populations of *Polyspora* sp*eciosa*, 5 populations of *Polyspora kwangsiensis*, 1 population of *Polyspora tonkinensis*, 2 populations of *Polyspora longicarpa*, 1 population of *Polyspora tiantangensis*, and 2 populations of *Polyspora hainanensis*. The investigation and collection of samples in this study have been approved by the local regulatory authorities. All voucher specimens were morphologically identified by Zhifeng Fan from Southwest Forestry University and deposited in the Herbarium of Southwest Forestry University.

### 2.2 Phenotypic measurement

**Figure 2:**
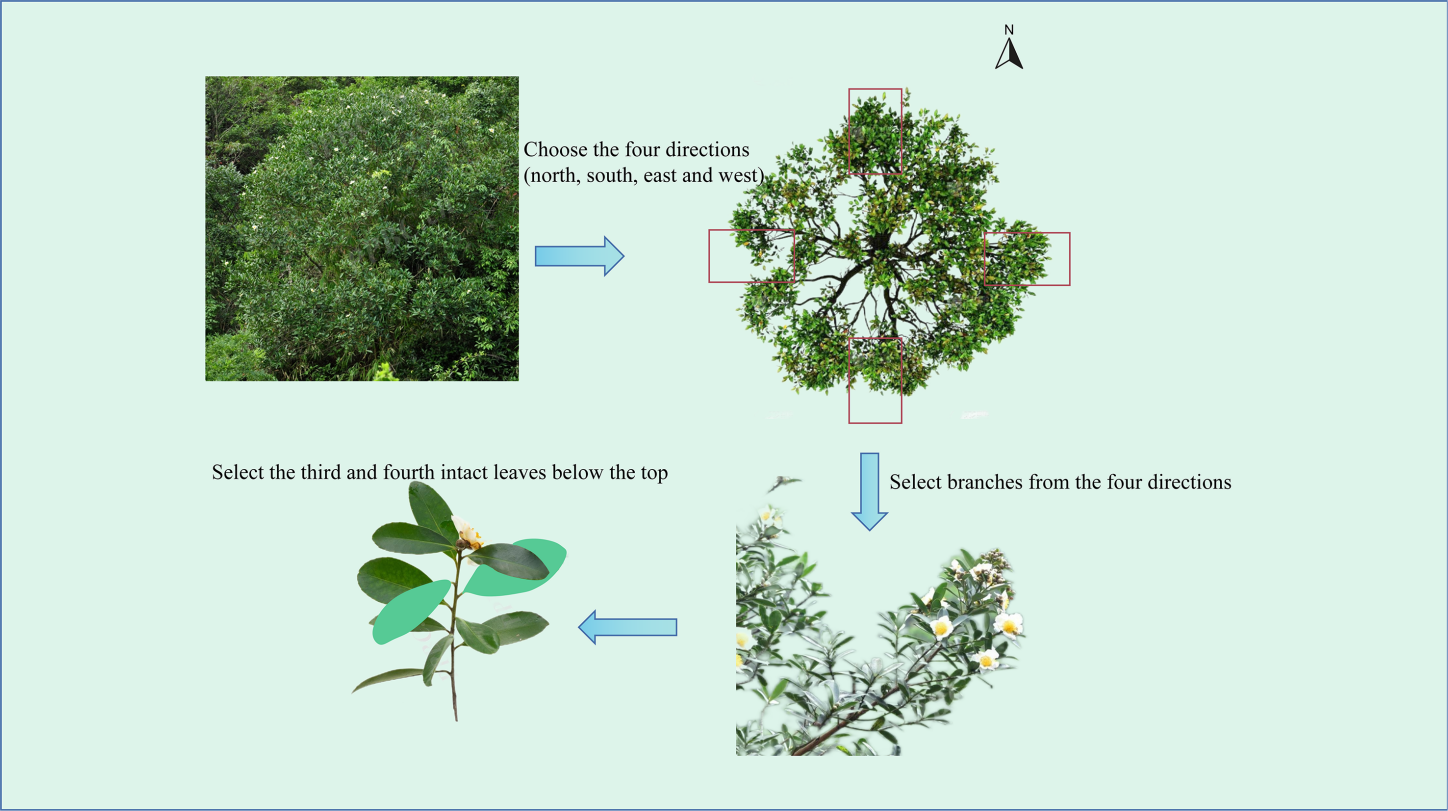
Schematic diagram of blade sampling.

A total of 31 indicators were utilized for phenotypic analysis (**Supplementary Table S2**), with 25 directly measured and 6 obtained through secondary calculations. For each individual plant, we selected different lateral branches from the main stem in each of the four cardinal directions (east, south, west, north). From each lateral branch, the 3rd to 4th fully developed mature leaves, counted from the top down, were chosen. (**Figure 2**). A total of 20 leaves were collected per plant, and indices such as leaf length, leaf width, and petiole length were measured. For each plant, 5 fully open flowers were randomly selected to measure floral traits such as flower diameter, petal length, and petal width. For each plant, 20 healthy mature fruits per plant were randomly collected and brought back to the laboratory to measure the fruit length, peduncle length, seed wing length and other seed and fruit data, and the fruit mass and seed mass were weighed. In the above measurements, the lengths, widths, and diameters are all measured via an electronic vernier caliper (measurement accuracy of 0.01 mm); and the weight is determined via an electronic balance (measurement accuracy of 0.01 g). Following scanning of the leaves via Digimizer software, parameters such as leaf area, leaf base angle as well as tip angles were measured accordingly (with measurement accuracies of 0.01 cm², 0.01cm and 0.01° respectively). The leaf shape index, petiole index, leaf roundness, fruit shape index, fruit stem index, and wing shape index are calculated via specific formulas.

Due to the influence of phenological period, flowering and fruiting patterns, and timing of field surveys for plants in the genus *Polyspora*, some populations exhibit insufficient or absent flowers, fruits, or seeds to meet the desired data volume. To ensure relative uniformity, we selected phenotypic data on floral organs from 16 populations (representing 4 species), fruit and seed data from 23 populations (covering all species), and leaf morphological data from all 32 populations.

### 2.3 Collection of environmental data

Geographical environment factors include 19 biological climate factors, 2 topographic factors, 2 soil complex factors, 6 ultraviolet radiation factors, 1 aridity index, 1 vegetation factor and 2 latitude and longitude factors (**Supplementary Table S3**). The bioclimatic factors data were obtained from the WorldClim database (https://www.worldclim.org). The soil composite factors were acquired from the Harmonized World Soil Database v 1.2, HWSD, (https://www.fao.org/soils-portal/soil-survey/soil-maps-and-databases/harmonized-world-soil-database-v12/en/). The ultraviolet radiation factor was sourced from the Global UV-B Radiation Database, gIUV, (https://www.ufz.de/gluv/). Environmental variable values for each population were extracted using the ArcGIS spatial interpolation method. Latitude, longitude, and altitude factors were selected based on GPS records collected during field surveys. Slope aspect was derived by analyzing altitude through ArcGIS 10.2 spatial analysis functions (https://www.esri.com/zh-cn/home). The vegetation data were downloaded from the Resource and Environmental Science Data Center of the Chinese Academy of Sciences (http://www.resdc.cn).

### 2.4 Morphological traits

By integrating both quantitative and qualitative morphological characteristics, a total of 15 morphological traits were carefully selected, comprising 7 vegetative traits and 8 reproductive traits. These traits are coded as either 0 for primitive or 1+ for evolved traits, with a few disordered traits being treated as equal distances (**Table 1**). The traits utilized and their corresponding codes are as follows:

**Table 1:** Selection and coding of morphological phylogenetic traits.

Code	Trait	Trait status and its coding
A	Spray	pubescence 0;Glabrous 1
B	Terminal bud	pubescence 0;Glabrous 1
C	Leaf length	≥14 cm 0;< 14 cm 1
D	Leaf petiole length	≤1cm 0;1-1.5 cm 1;1.5–2 cm 2;≥2 cm 3
E	Leaf back	pubescence 0;Glabrous 1
F	Leaf margin	Almost entire 0;Partially serrated 1;Serrated 2
G	Leaf texture	thick leathery 0;thin leathery 1
H	Flower diameter	≥8 cm 0;5–8 cm 1;<5 cm 2
I	Flower stalk length	≤3 mm 0;3–5 mm 1;≥5 mm 2
J	Style length	≤1 cm 0;1-1.5 cm 1;1.5–2 cm 2;≥2 cm 3
K	Sepal length	≤1 cm 0;1-1.5 cm 1;1.5-2cm 2;≥2cm 3
L	Bract number	6~7 piece 0;4~5 piece 1;2~3 piece 2
M	Ovary chamber number	6~8 chamber 0;5 chamber 1
N	Capsule length	≥3.5 cm 0;2.5-3.5 cm 1;≤2.5cm
O	Seed wing length	≤1.5 cm 0;1.5–2 cm 1;≥2 cm 2

### 2.5 Statistical analysis

The phenotypic data were entered and statistically analyzed using Excel. The characteristics and variation patterns of each phenotypic trait among and within populations were calculated by nested analysis of variance (ANOVA). Multiple comparisons were performed using the Duncan’s new multiple range method. The coefficient of variation for each phenotypic trait was determined by ANOVA.

The linear model for the nested ANOVA is as follows:


$$Y_{ijk}=L+S_{i}+T_{(i)j}+E_{(ij)k}$$

where $Y_{ijk}$ represents the kth observation of the jth individual of the ith population, L represents the total mean, $S_{i}$ represents the population effect (fixed), $T_{(i)j}\;$ represents the within-population monoculture effect (random), and $E_{(ij)k}$ represents the experimental error (Hao et al., 2017).

The phenotypic coefficient of variation is (CV) (%) =σ/ μ ×100 , where σ is the standard deviation and where μ is the mean.

The phenotypic differentiation coefficient.

$VST\;=\;(\delta_{t/s}^{2})/(\delta_{t/s}^{2}+\delta_{s}^{2})$ , where $\delta_{t/s}^{2}$ is the variance component between-population and $\delta_{s}^{2}$ is the within-population variance component (Ge et al., 1988).

Due to the potential strong autocorrelation of bioclimatic factors and ultraviolet factors within the research scale, an autocorrelation test was first conducted on the 33 geographical environmental factors. For pairs of environmental factors with a correlation coefficient |r| ≥ 0.8, only the factor with greater biological significance was retained, forming a more concise geographical environmental factor matrix (explanatory variable matrix) for each population. Due to the limited number of leaf phenotypic traits, we did not perform deduplications on these traits. All 10 leaf phenotypic factors were included in the analysis as the response variable matrix (**Supplementary Table S2**). Subsequently, redundancy analysis and correlation analysis were employed to examine the relationships between leaf phenotypic traits and geographical environmental factors. By summarizing the results of the two methods, the responsive relationships between phenotypic traits and environmental factors were explored. For the paired variables between leaf phenotypic traits and geographical environmental factors with a correlation coefficient |r| ≥ 0.6, regression analysis was conducted to explain the relationships between the phenotypic spatial variation in the genus *Polyspora* and geographical environmental factors.

Basis on the results of the phenotypic survey, morphological traits were encoded according to **Table 1**, and an interspecific branching data matrix table for the genus *Polyspora* was established. Using MrBayes, a phylogenetic Bayesian inference (BI) tree based on morphological data was constructed. The parameters were set to establish 4 Markov chains, with a random tree as the starting tree. The analysis was run for a total of 2,000,000 generations, with sampling every 100 generations. The first 25% of the samples were discarded, and a consensus tree was constructed from the remaining samples. Using Apterosperma oblata was used as the outgroup. We calculated the phylogenetic signal of morphological traits based on Blomberg’s K (Blomberg et al., 2003).

Cluster analysis, analysis of variance, redundancy analysis, regression analysis, morphological phylogenetic signals, and plotting were conducted using R software version 4.2.2.

## 3 Results

### 3.1 Leaf morphological diversity

The analysis of leaf phenotypic variability in *Polyspora* showed that among the domestic *Polyspora* species, the largest leaf blade was *P.* sp*eciosa*, with a mean leaf length of up to 227 mm, a leaf width of 64 mm, a leaf perimeter of 562 mm, and a leaf area of 8,718 mm^2^; the smallest leaf blade was *P. chrysandra*, with a leaf length of 123 mm, a leaf width of 42 mm, a leaf perimeter of 299 mm, and a leaf area of 3,504 mm^2^; and the leaves with greater roundness were *P. longicarpa* and *P. chrysandra* (**Table 2**). A greater roundness of leaves was found for *P. longicarpa* and *P. chrysandra*. The average coefficient of variation of leaf traits across different species ranges from 19.56% to 28.46% (**Table 2**). Specifically, *P. longicarpa* exhibits the largest degree of variation, while *P. tonkinensis* shows the smallest. With respect to individual traits, the highest degree of variation is observed in the leaf area of P. axillaris, reaching 52.46% (**Table 2**). In contrast, the smallest degree of variation is detected in the leaf roundness of *P. tiantangensis*, at 9.3% (**Table 2**). *P. hainanensis*, *P. kwangsiensis*, *P.* sp*eciosa*, *P. tiantangensis*, and *P. tonkinensi*s all demonstrate greater degrees of variation in leaf tips. Additionally, *P. axillaris*, *P. chrysandra*, and *P. longicarpa* display the greatest variation in leaf area, whereas they exhibit the smallest variation in leaf roundness.

**Table 2:** The leaf phenotypes characterization of *Polyspora.*.

Phenotypic traits	Units	*P. axillaris*		*P. chrysandra*		*P. hainanensis*		*P. longicarpa*		*P. tiantangensis*		*P.* sp*eciosa*		*P. kwangsiensis*		*P.tonkinensis*	
		Mean ± SD	CV(%)	Mean ± SD	CV(%)	Mean ± SD	CV(%)	Mean ± SD	CV(%)	Mean ± SD	CV(%)	Mean ± SD	CV(%)	Mean ± SD	CV(%)	Mean ± SD	CV(%)
PL Petiole length	mm	11.08 ± 3.58 f	32.31	9.05 ± 2.91 g	32.15	10.88 ± 3.36 f	30.88	14.05 ± 5.83 d	21.51	12.35 ± 3.88 e	41.49	21.91 ± 4.88 a	22.27	18.27 ± 3.93 b	31.42	16.64 ± 3.21 c	19.29
LL Leaf length	mm	136.60 ± 32.52 e	23.81	122.86 ± 25.13 f	20.45	161.68 ± 34.03 d	21.05	181.64 ± 42.93 c	17.01	175.33 ± 19.66 c	23.63	227.38 ± 40.08 a	17.63	203.54 ± 34.62 b	11.21	209.55 ± 25.10 b	11.98
LW Leaf width	mm	43.98 ± 12.56 e	28.56	42.44 ± 9.25 e	21.80	43.40 ± 9.02 e	20.78	70.03 ± 18.24 a	15.38	66.78 ± 12.80 ab	26.05	63.80 ± 12.26 bc	19.22	60.55 ± 9.31 c	19.17	50.91 ± 7.78 d	15.28
LSI Leaf shape index	–	3.19 ± 0.54 d	16.93	2.96 ± 0.59 e	19.93	3.75 ± 0.52 b	13.87	2.68 ± 0.58 f	15.88	2.70 ± 0.44 f	21.64	3.60 ± 0.44 b	12.22	3.40 ± 0.54 c	16.30	4.17 ± 0.55 a	13.19
PI Petiole index	–	0.08 ± 0.02 c	25.00	0.07 ± 0.02 de	28.57	0.07 ± 0.02 e	28.57	0.08 ± 0.02 cde	22.22	0.07 ± 0.02 e	25.00	0.10 ± 0.02 a	20.00	0.09 ± 0.02 b	28.57	0.08 ± 0.02 cd	25.00
LTA Leaf tip angle	∘	91.56 ± 26.25 a	28.67	76.43 ± 19.59 b	25.63	42.38 ± 19.70 e	46.48	69.55 ± 28.28 c	37.87	68.81 ± 26.04 c	40.66	36.39 ± 13.10 e	36.00	55.21 ± 20.91 d	37.84	37.38 ± 12.29 e	32.88
LBA Leaf base angle	∘	51.91 ± 18.06 c	34.79	66.69 ± 14.25 a	21.37	36.65 ± 13.01 e	35.50	61.31 ± 14.92 b	29.26	70.65 ± 15.66 a	24.34	46.45 ± 10.41 d	22.41	57.75 ± 16.90 b	22.17	44.34 ± 11.68 d	26.34
LP Leaf perimeter	mm	336.16 ± 79.93 f	23.78	298.90 ± 58.62 g	19.61	391.87 ± 82.85 e	21.14	469.98 ± 112.77 d	15.68	466.52 ± 59.19 d	23.99	562.3 ± 96.96 a	17.24	496.72 ± 77.91 c	12.69	521.29 ± 63.20 b	12.12
LA Leaf area	mm2	4082.70 ± 2141.77ef	52.46	3504.21 ± 1357.41f	38.84	4228.38 ± 1642.30e	38.84	8391.51 ± 3572.44ab	28.61	7670.59 ± 1991.27c	42.57	8718.17 ± 2780.33a	31.89	7743.34 ± 2215.05bc	25.96	6600.70 ± 1731.31d	26.23
LR Leaf roundness	–	0.43 ± 0.06 b	13.95	0.48 ± 0.06 a	12.50	0.34 ± 0.06 d	17.65	0.46 ± 0.07 a	15.38	0.43 ± 0.04 b	15.22	0.34 ± 0.04 d	11.76	0.39 ± 0.06 c	9.30	0.30 ± 0.04 e	13.33

The same lowercase letters following the morphological trait values for each species indicate that the trait shows no significant difference among species.

### 3.2 Flower morphological diversity

Due to the insufficient number of field observations and samples, floral phenotypic differentiation included only 16 populations of four species, namely, *P. chrysandra*, *P. hainanensis*, *P.* sp*eciosa* and *P. longicarpa*, and the remaining species did not have enough statistically significant floral organ phenotypic trait data. Phenotypic characterization of the flowers revealed that, among the domestically produced species, *P. longicarpa* had the largest flowers, with a mean flower diameter of 130.75 mm, a petal length of 69.72 mm, a petal width of 60.43 mm, and a sepal length of 15.89 mm. The smallest flowers were found on *P. hainanensis*, with a mean flower diameter of 41.32 mm, a petal length of 27.43 mm, a petal width of 24.92 mm, and a sepal length of 5.68 mm, the vast majority of the flowers were the same size as those on *P. hainanensis*. Most of the flowers were 6-merous, with an 8-merous variant found in *P. chrysandra* and a 5-merous variant found in *P. hainanensis*. The flowering phenotype of *P. chrysandra* plants was variable, with an average coefficient of variation of 16.26%, and the most variable trait was petal length (24.56%). Except for the number of petals, the petal width of the *P.* sp*eciosa* plants was the most stable, with a coefficient of variation of 6.84% (**Table 3**).

**Table 3:** Flower phenotypes characterization of *Polyspora.*.

Phenotypic trait	Units	*P. chrysandra*		*P. hainanensis*		*P. longicarpa*		*P.* sp*eciosa*	
		Mean ± SD	CV(%)	Mean ± SD	CV(%)	Mean ± SD	CV(%)	Mean ± SD	CV(%)
FD Flower diameter	mm	68.80 ± 9.71 c	14.11	41.32 ± 2.90 d	7.02	130.75 ± 12.34 a	9.44	91.63 ± 6.64 b	7.25
PEL Petal length	mm	35.42 ± 8.70 c	24.56	27.43 ± 2.65 d	9.66	69.72 ± 8.41 a	12.06	48.11 ± 3.29 b	6.84
PEW Petal width	mm	28.91 ± 3.37 c	11.66	24.92 ± 2.48 d	9.95	60.43 ± 7.91 a	13.09	38.85 ± 3.97 b	10.22
PEN Petal number	–	6.10 ± 0.45 a	7.38	5.90 ± 0.31b	5.25	6.00 ± 0.00 ab	0.00	6.00 ± 0.00 ab	0.00
SL Sepal length	mm	8.22 ± 1.94 b	23.60	5.68 ± 0.45 c	7.92	15.89 ± 2.45 a	15.42	9.71 ± 1.69 b	17.40

The same lowercase letters following the morphological trait values for each species indicate that the trait shows no significant difference among species.

### 3.3 Fruit and seed morphological diversity

For the analyses of fruit and seed phenotypic traits, only 18 groups of five species were included, again due to insufficient sample data, *P. axillaris*, *P. chrysandra*, *P. hainanensis*, *P. longicarpa*, *P.* sp*eciosa*, respectively. The largest fruit and heaviest seed among the domestic species of *Polyspora* was *P. longicarpa*, with a mean capsule length of 39.75 mm, a thousand-seed weight of 42 g, and a wing length of 14.92 mm; the species with smaller seeds were *P. hainanensis* and *P. axillaris*, with capsule lengths of 22.85 mm and 23.94 mm, respectively, and thousand- seed weights of 12 g and 11 g, respectively, and the shortest seed wings were those of *P. axillaris*, with a mean value of 8.55 mm. The coefficient of variation for the seed phenotype was the highest for *P. axillaris* (20.15%), and the smallest was for *P. hainanensis* (14.16%). Among the traits, wing length had the greatest variation, with an average coefficient of variation of 21.05%, and capsule length was the most stable (12.75%). The seed weight of *P. axillaris* had the greatest variation, with a coefficient of variation of 36.36%. The wing width of *P. hainanensis* had the smallest variation, with a coefficient of variation of 9.59% (**Table 4**).

**Table 4:** The fruit and seed phenotypes characterization of *Polyspora.* .

Traits	Units	*P. axillaris*		*P.chrysandra*		*P.hainanensis*		*P.longicarpa*		*P.speciosa*	
		Mean ± SD	CV (%)	Mean ± SD	CV (%)	Mean ± SD	CV (%)	Mean ± SD	CV (%)	Mean ± SD	CV (%)
CL Capsule length	mm	23.94 ± 3.27 d	13.66	30.47 ± 4.07 b	13.36	22.85 ± 2.36 d	10.33	39.75 ± 4.51 a	11.35	26.95 ± 4.06 c	15.06
CSL Capsule stem length	mm	8.16 ± 1.38 d	16.91	8.96 ± 2.62 c	29.24	9.11 ± 1.02 c	11.20	14.71 ± 2.14 a	14.55	10.21 ± 1.95 b	19.10
CSTI Capsule stem index	–	0.34 ± 0.05 c	14.71	0.29 ± 0.08 d	27.59	0.40 ± 0.06 a	15.00	0.37 ± 0.07 b	18.92	0.39 ± 0.08 ab	20.51
SW Seed weight	g	0.11 ± 0.04 d	36.36	0.23 ± 0.05 b	21.74	0.12 ± 0.02 d	16.67	0.42 ± 0.03 a	7.14	0.17 ± 0.03 c	17.65
ST Seed thickness	mm	1.52 ± 0.28 c	18.42	1.61 ± 0.28 bc	17.39	1.63 ± 0.31 b	19.02	1.75 ± 0.23 a	13.14	1.74 ± 0.29 a	16.67
SWL Seed length (include wing)	mm	12.85 ± 2.47 d	19.22	19.25 ± 2.47 b	12.83	16.33 ± 2.50 c	15.31	21.67 ± 3.56 a	16.43	17.24 ± 3.55 c	20.59
WL Wing length	mm	8.55 ± 2.00 d	23.39	12.73 ± 2.44 b	19.17	11.17 ± 2.02 c	18.08	14.92 ± 2.91 a	19.50	12.10 ± 3.04 b	25.12
WD Wing width	mm	4.39 ± 0.98 c	22.32	5.40 ± 0.72 b	13.33	4.69 ± 0.45 c	9.59	7.03 ± 0.79 a	11.24	5.68 ± 0.98 b	17.25
WSI Wing shape index	–	1.96 ± 0.32 c	16.33	2.38 ± 0.45 a	18.91	2.37 ± 0.29 a	12.24	2.14 ± 0.44 b	20.56	2.14 ± 0.43 b	20.09

The same lowercase letters following the morphological trait values for each species indicate that the trait shows no significant difference among species.

### 3.4 Population differentiation of phenotypic traits within the genus

To explore the ratio of variance components to variation for each trait, we calculated the nested variance component composition ratio. The results showed that the phenotypic differentiation coefficients of each trait exhibited a wide range of variation, from 1.49% to 97.14%, among the domestically produced species of *Polyspora*, with 0% or 100% for individual traits due to an insufficient amount of data (**Supplementary Table S4**). The traits that were not listed for each species were missing data; only one population was investigated for *P. tonkinensis* and *P. tiantangensis*, and no population differentiation study was carried out. A higher coefficient of variation indicates a greater degree of trait dispersion and higher phenotypic diversity within the population. The average phenotypic differentiation coefficient was 66.87% for *P. axillaris*, 40.77% for *P. chrysandra*, 40.31% for *P. hainanensis*, 48.18% for *P.* sp*eciosa*, 35.11% for *P. kwangsiensis*, and 5.02% for *P. longicarpa*. The phenotypic differentiation between populations was greater than that within populations for *P. axillaris.* For *P. chrysandra*, inter-population phenotypic differentiation was greater than intra-population differentiation. For *P.* sp*eciosa*, the phenotypic differentiation between inter-population and intra-population was not obvious. For *P. hainanensis*, *P. kwangsiensis*, *and P. longicarpa*, the primary variation originates from within populations. Among the traits, the phenotypic differentiation coefficients for leaf length were generally small, and those for wing length, wing width, and seed weight were large.

### 3.5 Correlations between leaf phenotypes and geoenvironmental factors in *Polyspora*

**Figure 3:**
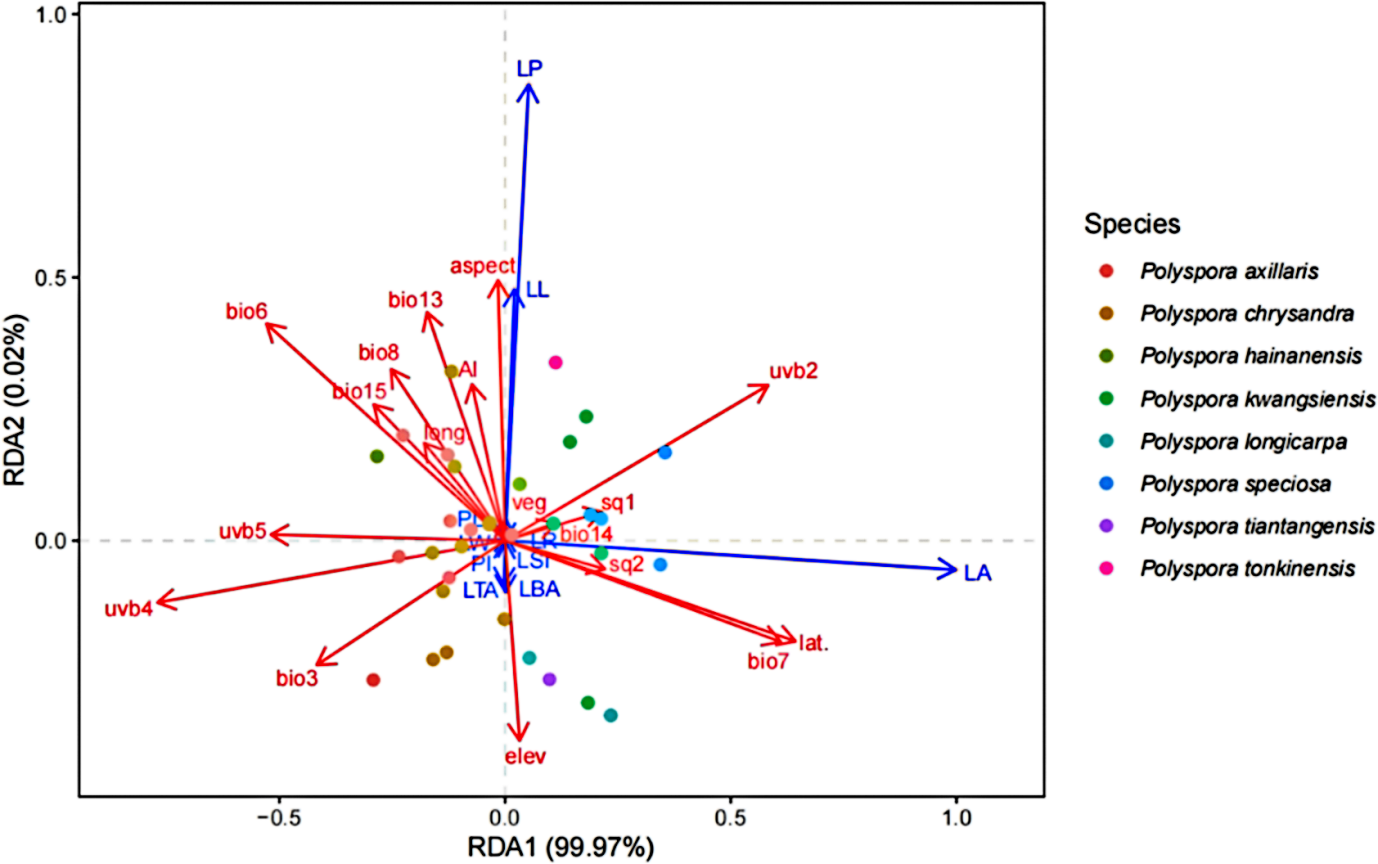
RDA of leaf phenotypic traits and geographic environmental factors in *Polyspora*. Environmental factors used in RDA analysis include: (bio03)Isothermality, (bio06) Min Temperature of Coldest Month, (bio07)Temperature Annual Range, (bio08) Mean Temperature of Wettest Quarter, (bio13) Precipitation of Wettest Month, (bio14) Precipitation of Driest Month, (bio15) Precipitation Seasonality, (uvb2) Seasonal variation of ultraviolet radiation, (uvb4) Minimum monthly average UV, (uvb5)sum of maximum monthly UV, (Elev) Elevation, Aspect, (sq1)Soil nutrient availability, (sq2)Soil nutrient retention capacity, (AI)Aridity index, (Veg) Vegetation types, (long.) Longitude, (lat.)Latitude.

Through the autocorrelation analysis of 33 geoenvironmental factors, 18 environmental factors were ultimately retained to form a matrix of explanatory variables. The results of the RDA of the two matrices showed (**Figure 3**) that the first two sorting axes basically explained all the leaf phenotypic variation (cumulative explanation degree 99.99%). The degree of explanation of each type of factor was as follows: bioclimatic factor (42.24%) > UV factor (33.14%) > geographic factor (14.57%) > soil factor (7.73%) > drought factor (1.30%) > topographic factor (0.85%) > vegetation factor (0.17%). Among the individual geoenvironmental factors, the factor with the most explanations is latitude (lat.), annual temperature difference (bio7), seasonal variation in ultraviolet radiation (uvb2), lowest monthly mean ultraviolet radiation (uvb4), coldest monthly mean temperature (bio6), and the sum of the highest seasonal monthly mean ultraviolet radiation (uvb5), which are the six indicators that have the greatest influence on Axis 1, with correlation coefficients |r| exceeding 0.5. Among them, latitude, annual temperature difference, and seasonal variation in UV radiation were strongly positively correlated with leaf area, and the sum of the lowest monthly mean UV radiation, the coldest monthly mean temperature, and the highest seasonal monthly mean UV radiation were strongly negatively correlated with leaf area. In the four quadrants, the intraspecific populations of each species were more aggregated, and the selection of different species on geoenvironmental factors showed more obvious species differentiation, suggesting that the species’ own genetic factors also had a greater influence on leaf phenotypes.

### 3.6 Geographic clustering of phenotypic traits in the *Polyspora*

**Figure 4:**
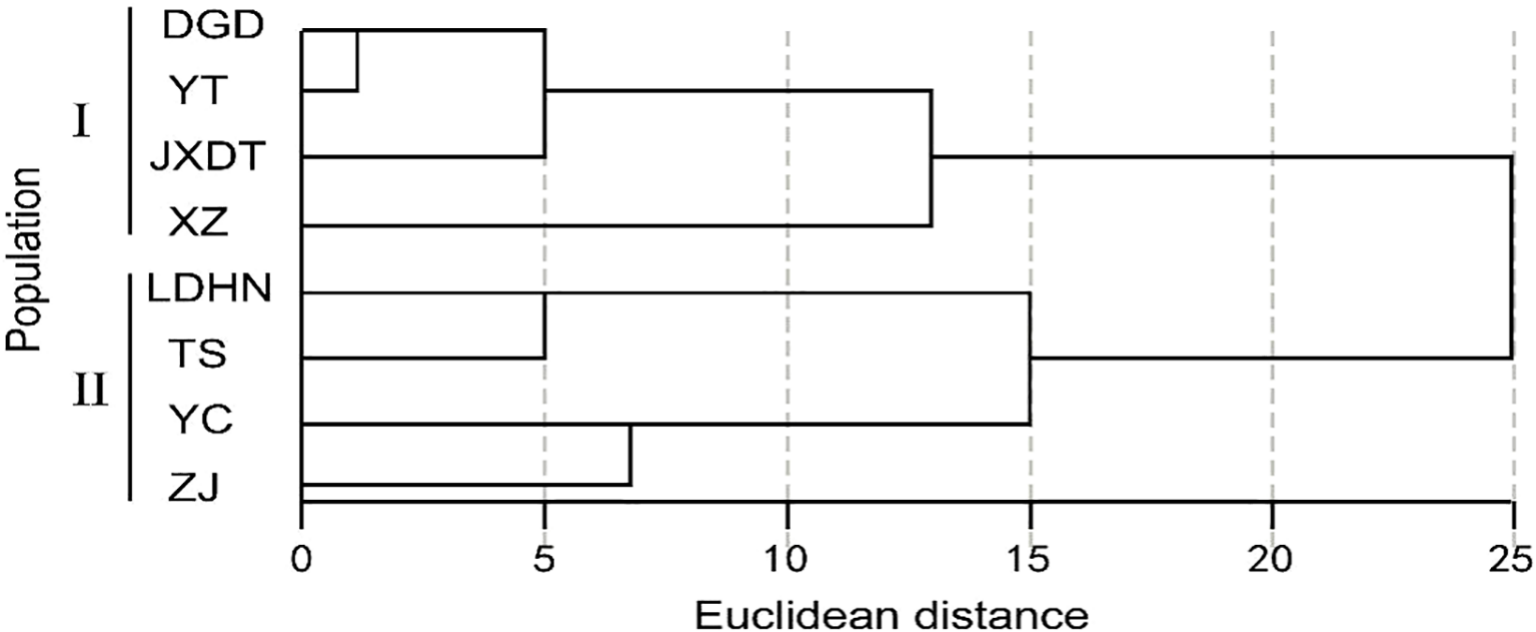
Clustering map of *P. axillaris* populations based on leaf, fruit and seed traits. The letters represent the colony codes of the collection sites, which correspond to the site information listed in **Supplementary Table S1**.

Geographic clustering analysis was carried out on 8 P*. axillaris* plant populations based on 19 leaf and seed phenotypic traits (**Figure 4**). At an Euclidean distance of 15, the eight populations were partitioned into two distinct branches. In particular, the Dangan Island Zhuhai Group (DGD), the Yantian Group of Shenzhen (YT), the Jiexi Group of Jieyang City (JXDT), and the Xiangzhou Group of Zhuhai City (XZ) were clustered into Branch I. Correspondingly, the Ledong Group of Hainan Province (LDHN), the Taishan Group of Jiangmen City (TS), the Yangchun Group of Yangjiang City (YC), and the Zijin Group of Heyuan City (ZJ) were clustered into Branch II. The results of geographical clustering analysis of 8 *Polyspora* populations were generally consistent with the longitudinal variation trends of leaf and seed phenotypic traits. Clade I is located in the eastern part of Guangdong, Province, which has larger longitudes and relatively smaller leaves. Clade II, with the exception of the Zijin group, is distributed in the western part of Guangdong Province, which exhibits smaller longitudes and relatively larger leaves.

**Figure 5:**
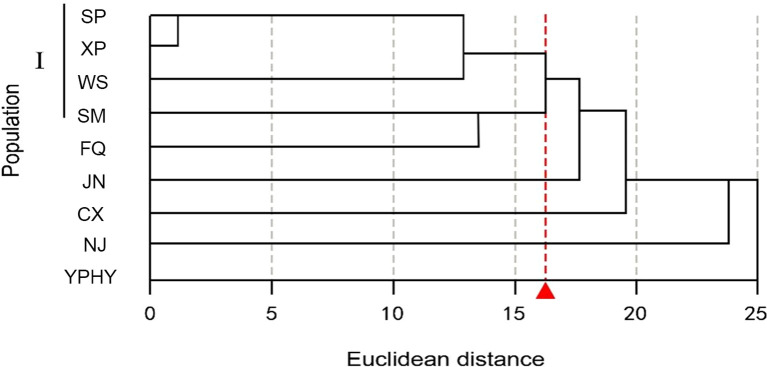
Geographical cluster analysis of *P. chrysandra* based on phenotypic traits. The letters represent the colony codes of the collection sites, which correspond to the site information listed in **Supplementary Table S1**.

The geographical clustering of *P. chrysandra* based on 31 phenotypic traits reveals that, at an Euclidean distance of 16, five populations in south-central Yunnan (Shiping, Xinping, Wenshan, Simao, Fengqing) are clustered into one group, whereas the other five populations do not cluster according to geographical distribution (**Figure 5**). It is indicated that there exists discontinuity in the overall phenotypic variation of *P. chrysandra*, and the phenotypic traits of populations in central, southeastern, and southern Yunnan are relatively similar.

Geographic clustering analyses of phenotypic traits were not performed for the remaining six species due to the small number of populations and close proximity of *P. hainanensis*, *P. longicarpa*, *P. tiantangensis*, *P.* sp*eciosa*, *P. kwangsiensis* and *P. tonkinensis*.

### 3.7 Morphology-based phylogenetic analyses of the Chinese *Polyspora*

**Figure 6:**
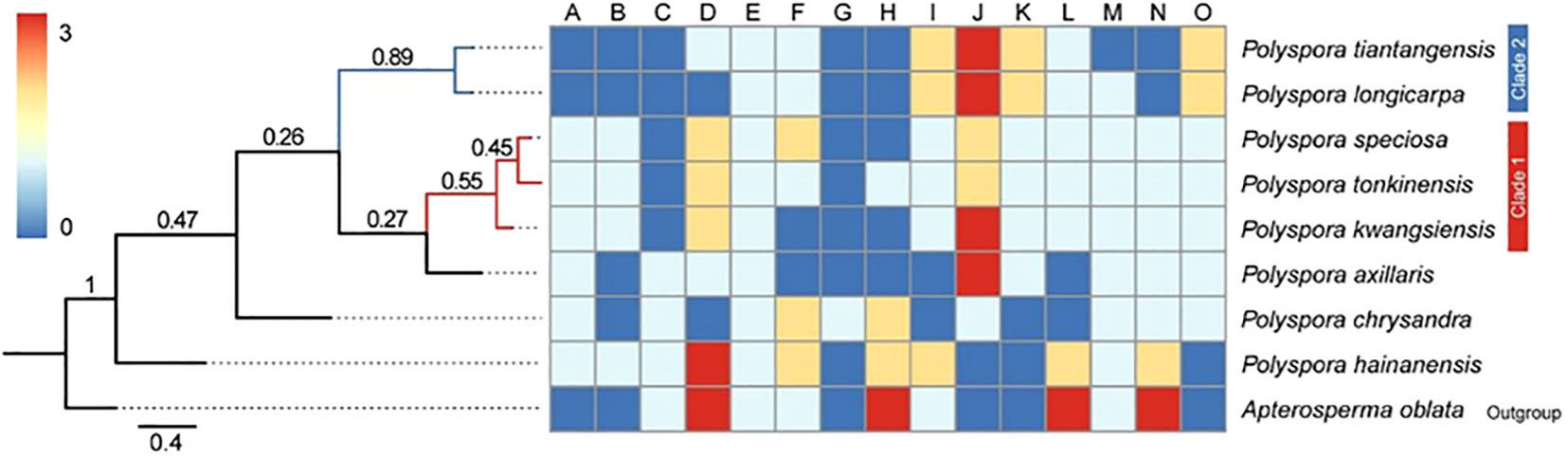
Phylogeny of the Chinese *Polyspora* based on morphology. **(A–O)** are the codes for each morphological trait. **(A)** Spray, **(B)** terminal bud, **(C)** leaf length, **(D)** Leaf petiole length, **(E)**Leaf back, **(F)** Leaf margin, **(G)**Leaf texture, **(H)** Flower diameter, **(I)** Flower stalk length, **(J)** style length, K sepal length,**(L)**bract number, **(M)** ovary chamber number, **(N)** capsule length, **(O)** seed wing length.

The Bayesian phylogenetic tree based on 15 morphological traits showed that two nodes were strongly supported, with the basal taxon being *P. hainanensis*, the tropical component of the *Polyspora* in China, *P.* sp*eciosa*, *P. tonkinensis*, and *P. kwangsiensis*, with the three species forming a weak sister (Clade 1); *P. tiantangensis* and *P. longicarpa*, diverged later and formed a sister relationship between the two (Clade 2); and all the other nodes of the morphological phylogenetic tree presented a visual rate of less than 0.5 (**Figure 6**).

The K values of phylogenetic signal strengths for 15 morphological traits of 8 domestically produced species of the *Polyspora* were calculated by the Blomberg method, and the results are shown in **Table 5**. Significant phylogenetic signals were detected for style length, sepal length, capsule length, and wing length (K>>1, *p* < 0.05); flower diameter, leaf length, and bract number also had some spectral signals.

**Table 5:** Phylogenetic signals of the morphological traits of *Polyspora.*.

phenotypic trait	K	*pv*alue-K	K.star	*pv*alue-K.star	Lambda	*pv*alue- Lambda
A	0.8813	0.0540	1.1376	0.0480	1.0376	0.0010
B	0.5398	0.0610	0.9351	0.0610	1.0375	0.0010
C	1.2467	0.0270	1.2235	0.0480	1.0376	0.0010
D	0.8825	0.0130	0.8388	0.0230	0.9831	0.0073
E	0.8982	1.0000	0.0000	1.0000	0.7448	NA
F	0.1385	0.6780	0.2787	0.5080	0.0001	1.0000
G	0.4653	0.2240	0.9548	0.1150	1.0376	0.0010
H	1.8862	0.0050	1.1437	0.0130	0.9573	0.0010
I	0.4201	0.1110	0.8774	0.0150	1.0375	0.0010
J	2.2614	0.0020	1.5545	0.0040	1.0285	0.0010
K	2.3019	0.0030	2.1184	0.0010	1.0376	0.0010
L	1.8798	0.0040	1.1121	0.0080	1.0376	0.0010
M	0.0952	0.6530	0.1804	0.6460	0.0001	1.0000
N	3.3716	0.0010	1.8785	0.0010	1.0376	0.0010
O	2.8465	0.0080	2.1813	0.0010	1.0376	0.0010

## 4 Discussion and conclusion

### 4.1 Characteristics of phenotypic variation in *Polyspora*

**Figure 7:**
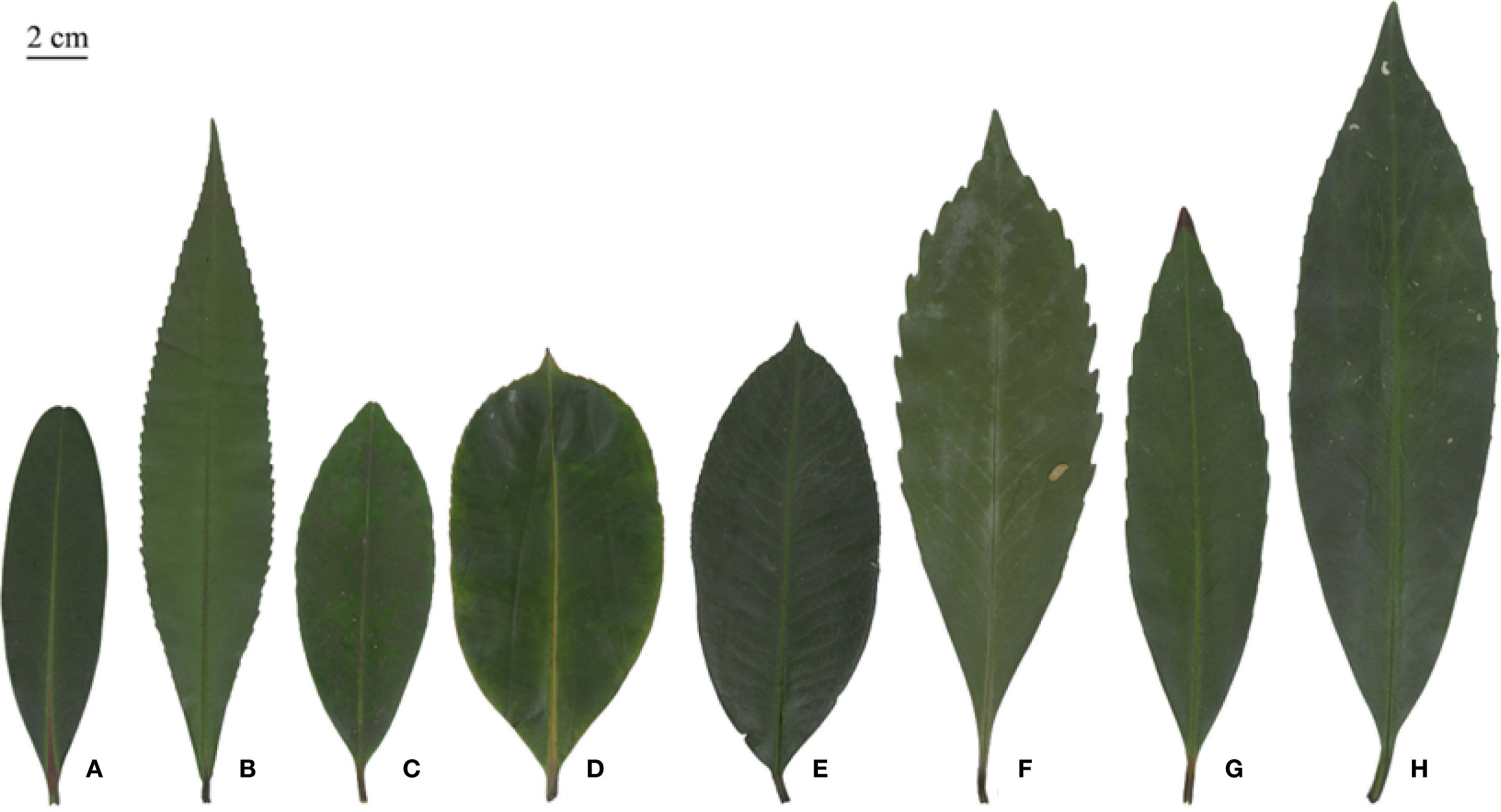
Typical leaf morphology of *Polyspora* species. **(A)** *P. axillaris*, **(B)** *P. hainanensis*, **(C)** *P. chrysandra*, **(D)** *P. longicarpa*, **(E)** *P. tiantangensis*, **(F)** *P.* sp*eciosa*, **(G)** *P. tonkinensis*, **(H)** *P. kwangsiensis.* All leaf images are original photographs of experimental samples.

Phenotypic traits are an important basis for identifying germplasms, conserving species diversity and selecting and breeding new varieties (Duan, 2020), maintaining both stability and variability (Zhang et al., 2019), and being affected by both the species’ own genetic material and the ecological environment (Wang et al., 2019). In general, a coefficient of variation of more than 10% is considered to indicate a large trait difference (Li et al., 2019b; Dong et al., 2020). Multivariate statistical analyses of a total of 31 traits of leaves, flowers, fruits and seeds of eight species of the *Polyspora*, showed that the species of *Polyspora* differed significantly between and within species, and were rich in phenotypic variability. The mean coefficient of variation for leaf traits was 24%, that for flower phenotypes was 11.98%, and that for fruit and seed traits was 17.49%; these values were all greater than 10%. Among the leaf traits, the coefficient of variation for the leaf apical angle was the highest, with a mean value of 35.76%. The leaf shape of the Polyspora shows a high variability (obovate, lanceolate, oblong, narrow-oblong, etc.). The leaf tip is strongly affected by the leaf shape, which manifests as acuminate, acute, slightly concave, convex, rounded or obtuse among the different species and groups; this is the main factor leading to the large variation in the angle of the tip of the leaf (**Figure 7**). Typically, phenotypic variation in nutritional traits is more influenced by the environment than that in reproductive traits, which are relatively stable. A comparison of the phenotypic variation among leaves, flowers and fruits revealed that the results of the present study also confirmed this pattern. The capsule is cylindrical and dehiscent at maturity, and the seed has a terminal wing that exceeds the length of the seed itself; these are the most obvious characteristics that distinguish the *Polyspora* from the rest of Theaceae.

The results of the phenotypic differentiation study showed that the phenotypic differentiation of most species of *Polyspora* occurred mainly within the population. The fact that insect-pollinated plants have shorter pollen dispersal distances compared to wind-pollinated plants, making intergroup gene exchange more difficult, may explain the constraints on intergroup genetic differentiation. This leads to the phenomenon where most species are predominantly phenotypically differentiated within their groups. In addition, the seeds of this genus are flattened and winged in the upper part, and the seeds are lighter; these conditions are suitable for wind propagation, and, to a certain extent, can also promote intergroup gene exchange, thus leading to a greater degree of intergroup differentiation in some species. The phenotypic differentiation of *P. axillaris* mainly occurs via intergroups, and the nonsignificant differences in phenotypic differentiation between and within *P.* sp*eciosa* provide good evidence of this feature.

**Figure 8:**
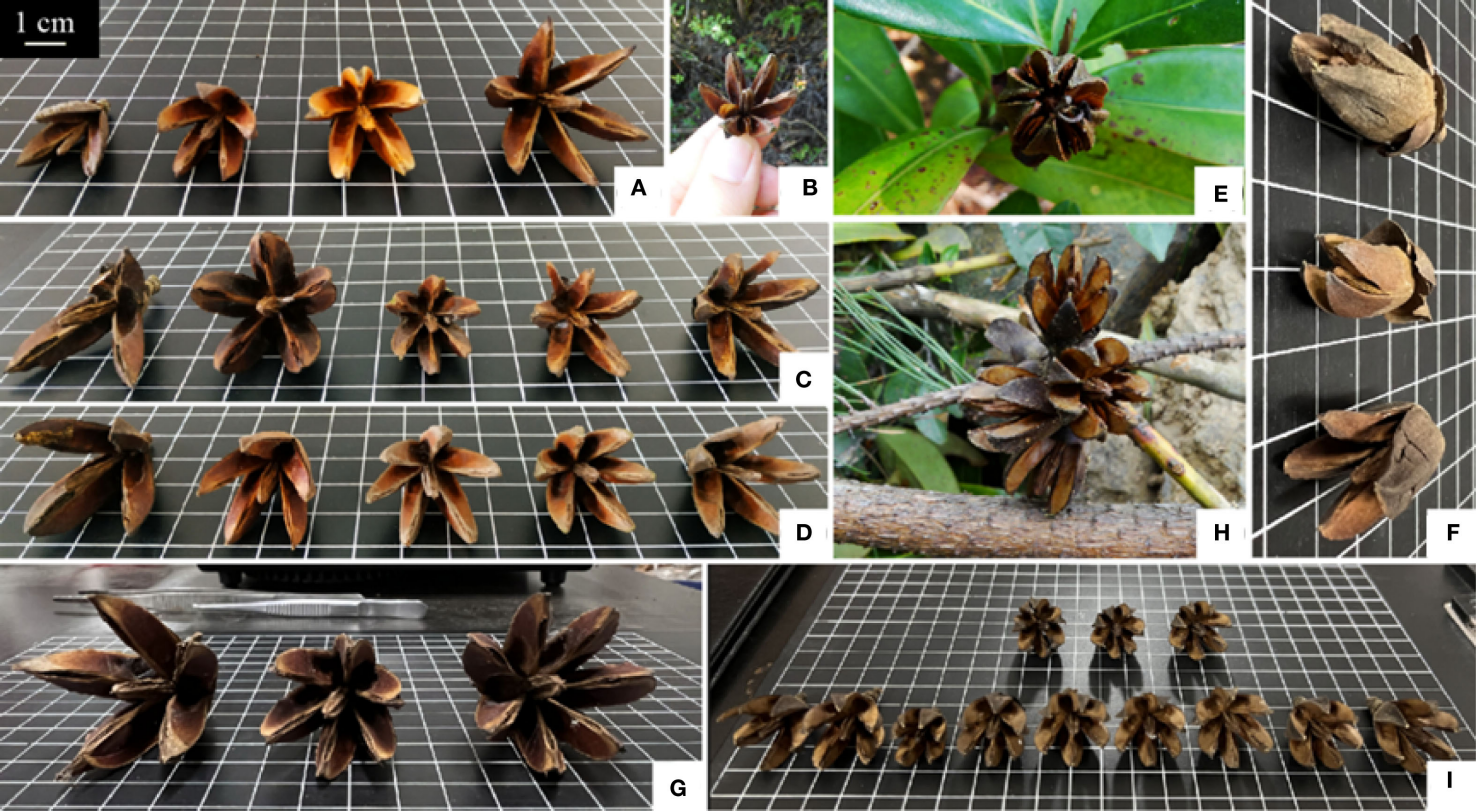
Capsule dehiscence of *Polyspora.* ***(*A*)*** *P. axillaris* in Dangan island, Zhuhai city, **(B)** *P. axillaris* in Meishajian, Shenzhen city, **(C)** *P. axillaris* in Jianyang county, Jiexi city, **(D)** *P. axillaris* in Fenghuang mountain, Zhuhai city, **(E)** *P. chrysandra* in Jinning county, Kunming city, **(F)** *P. chrysandra* in Yongping county, Dali city, **(G)** *P. longicarpa* in Yongping county, Dali city, (h/i) *P.* sp*eciosa* in Jinxiu county, Laibin city; Base plate grid is 1 cm×1 cm. All Capsule dehiscence of *Polyspora* images are original photographs of experimental samples.

The ovaries of *Polyspora* are usually 5-loculed, and the capsule splits into 5 valves at maturity. The main characteristic of *P. tiantangensis* that distinguishes it from *P. longicarpa* is that the ovary contains 6–8 cells (Deng and Fan, 1999). During field surveys, *P. longicarpa* capsules with 3, 4 and 6 valves were found on Dangang Island (DGD), Fenghuang Mountain (XZ) and Dabei Mountain (JXDT); *P. chrysandra* capsules with 3, 4 and 6 valves were found in Jinning District of Kunming City (JN) and Yongping County of Dali Prefecture (YPHY); and *P. longicarpa* capsules with 6 and 7 valves were found on Baotai Mountain of Yongping County of Dali Prefecture (YPCG). In particular, the *P.* sp*eciosa* group in Jinxiu County, Laibin city, Guangxi Province (JXSC), has 6- and 7-valved capsules, whereas conventional 5-valved capsules are rare (**Figure 8**). Among the 4,600 fruits observed in 23 populations of *Polyspora*, 5-lobed fruits composed the overwhelming majority of the fruits, but 3, 4, 6, 7, and 8 lobed fruits were produced by different species. These findings are not unique to *P. tiantangensis*, and the reasons for the differentiation of ovaries and the number of fruits with 5-lobed fruits need to be further investigated. The results of both leaf and fruit morphology studies support the idea that *P. tiantangensis* is an intraspecific variant of *P. longicarpa*, but as molecular biology has become an essential tool in plant taxonomy, the relationship between the two species needs to be further determined in combination with subsequent molecular data.

### 4.2 Environmental adaptations of leaf phenotypic traits in the *Polyspora*

Plant traits are the result of interactions between gene expression and environmental factors over a long period of evolution (Osada et al., 2015). A phenotype is a combination of various morphological traits and is a direct expression of biological genetic variation (Rosique-Esplugas et al., 2022; Yuan et al., 2023). Changes in growth space and the environment can promote phenotypic adaptive responses in species (Morales et al., 2018; Neves et al., 2020). When phenotypes are associated with environmental gradients, these responses are often elicited by local adaptation, phenotypic plasticity, etc., and can influence gene flow and distribution patterns (Wanderley et al., 2018). Nutritional traits are often subject to greater environmental selection pressures than reproductive traits. Leaves are the main organs of plants for photosynthesis and material production, they have the largest contact area with the external environment, and are the most sensitive to environmental changes (Xu et al., 2023). Leaf traits are the product of long-term adaptive evolution of plants in specific habitats, they directly affect the basic functions of plants and directly reflect their survival strategies (Klich, 2000), and are often used to monitor environmental changes (Ram et al., 2015).

Plant leaf shape and size may have different patterns of variation across taxa, with shape generally being conserved and variation usually caused by its own genetic material; however, size is considered to be highly variable, and variation is influenced mainly by the environment (i.e., phenotypic plasticity) (Chitwood et al., 2013; Maestri et al., 2016). Leaves of plants in arid, high-latitude, and high-altitude regions are small in size, whereas those in high-humidity, hot, and sunny regions are large in size (Wright et al., 2017). Among the 10 leaf morphological indices used in this study, 5 reflect leaf shape and the remaining 5 reflect leaf size. Specifically, the indices related to leaf shape include leaf shape index, petiole index, leaf base angle, leaf tip angle, and leaf roundness, while the remaining 5 indices are associated with leaf size. The average coefficient of variation of leaf shape was 23.61%, the average coefficient of variation of leaf size was 24.40% in *Polyspora* domestica, and the leaf phenotypic variation of each species was influenced by its own genetic factors and geographical environment.

Plants in unfavorable environments improve their ability to access resources by moderately altering leaf morphology (Feng et al., 2021). Among the geoenvironmental factors, isothermality (bio3), the lowest monthly mean UV radiation (uvb4) and the sum of the highest seasonal monthly mean UV radiation (uvb5) were significantly negatively correlated with most of the leaf phenotypic indices of *Polyspora*. These findings reflect the resistance response and environmental adaptation of *Polyspora* leaves and the small size of *Polyspora* leaves in areas with strong UV radiation, relative dryness, and coldness. Latitude (lat.), seasonal variation in ultraviolet radiation (uvb2), and annual temperature range (bio7) exhibited a significant positive correlation with most leaf phenotypic traits. This unusual pattern is primarily attributed to the four populations of *P. speciosa* distributed in the vicinity Chongqing. These populations are distributed at the highest latitudes, with the most significant variations in temperature and ultraviolet radiation. Furthermore, *P.* sp*eciosa* possesses the largest leaves among domestic *Polyspora* species, a feature that contributes to this phenomenon. This characteristic is presumably determined by the species’ inherent genetic material. Temperature factors such as isothermality (bio3), mean coldest monthly temperature (bio6) and annual temperature difference (bio7) were strongly correlated with leaf traits, whereas rainfall-related factors were not strongly correlated with leaf shape. In addition to rainfall, temperature was the key factor influencing the variation in leaf traits in *Cunninghamia lanceolata*, a widely spread timber tree species in southern China (Xu et al., 2023). These findings are consistent with the results of studies of fir (*Cunninghamia lanceolata*), a widely distributed timber tree species in southern China, and *Litsea coreana* var. *sinensis*, which is also a dominant component of broadleaf evergreen forests (Pan et al., 2020). Generally, most of the bioclimatic factors were not strongly correlated with the leaf phenotypes of *Polyspora*, and the leaf phenotypic variation in *Polyspora* was the result of the joint influence of its own genetic material and geoenvironmental factors; moreover, the two types of factors played comparable roles.

Plants are often exposed to incident photons of ultraviolet radiation (UV-B, wavelength 280–315 nm) when they are searching for light for photosynthesis (Shinkle et al., 2022). Although these photons represent only 0.5% of the solar radiation that reaches the biosphere (Blumthaler, 1993), UV radiation has a significant effect on plants, causing morphological changes in plants (Jenkins, 2009). UV radiation rapidly induces gene regulation, leading to cumulative changes in plant physiology and morphology, but the resulting morphological changes are slow, suggesting that the transgenerational effects of solar shortwave UV radiation may contribute to plant adaptation through morphological traits (Yan et al., 2020). Leaves are considered the most important target organ for UV radiation in plants (Verdaguer et al., 2012). Plants protect sensitive tissues from UV-B radiation by reflecting UV photons from the leaf surface by thickening the cuticle, waxy layer, trichomes and altering the optical properties of the cells and other structures (Jenkins, 2009; Robson et al., 2019). In this study, UV radiation ranked second among the factors explaining the association between UV radiation and leaf phenotypes. Additionally, the sum of the lowest monthly mean UV radiation (uvb4) and the highest seasonal monthly mean UV radiation (uvb5) showed a significant negative correlation with most leaf morphological traits, indicating that the leaves of *Polyspora* species are highly sensitive to UV radiation. Most of the species in the strong UV-B radiation zone had thick leathery leaves, suggesting that *Polyspora* adapts to UV radiation stress mainly by changing the structure of the leaf epidermis to protect chloroplastic tissues. RDA and CA showed that leaf area was strongly negatively correlated with two UV factors (uvb4 and uvb5), i.e., the leaf area decreased with increasing UV radiation, which was supported by the fact that the leaf area of six woody species of Mediterranean sclerophyllous evergreen forests was negatively correlated with the sum of the highest seasonal monthly mean UV radiation (uvb5). This phenomenon was confirmed by physiological experiments with six woody plants from a Mediterranean sclerophyllous evergreen forest, which showed that UV radiation leads to an increase in the thickness of leaf fenestration tissue, a reduction in leaf area, and an increase in leaf weight per unit area, which in turn protects photosynthetic structures and nucleic acids from damage (Verdaguer et al., 2012).

In addition to the leaf phenotype, the body size of plants in *Polyspora* is also good for adapting to the environment. In tropical rainforests and monsoon evergreen broad-leaved forests, *P. axillaris* can grow into trees up to 10 m high, whereas on the windward slopes of Zhuhai Gaolan Island and Tandang Island, *P. axillaris* can transform into low bushes due to the influence of typhoons, which is a better reflection of phenotypic plasticity.

Due to the limitations of material and data volume, this study explored only the relationships between leaf morphological traits and geographic environmental factors, and preliminarily revealed the geographic variation pattern of leaf phenotypes in the *Polyspora*, as well as the main environmental factors affecting leaf phenotypic variation. However, the adaptive evolution of leaves is achieved mainly by leaf function. Subsequent studies should investigate the physiological traits (e.g., photosynthetic capacity, dark respiratory capacity), structural traits (e.g., fenestrated tissues, specific leaf weight), and chemical traits (e.g., nitrogen and phosphorus contents) of leaves in *Polyspora* species. Such studies would aim to link leaf morphological traits with functional traits, analyze the leaf economic spectrum of *Polyspora* species, explore the relationships between leaf functional traits and the environment, and ultimately elucidate the evolution of leaf adaptive strategies and the mechanisms underlying their interaction with the environment.

## Data Availability

The original contributions presented in the study are included in the article/**Supplementary Material**. Further inquiries can be directed to the corresponding author.
